# Impact of QTL properties on the accuracy of multi-breed genomic prediction

**DOI:** 10.1186/s12711-015-0124-6

**Published:** 2015-05-08

**Authors:** Yvonne CJ Wientjes, Mario PL Calus, Michael E Goddard, Ben J Hayes

**Affiliations:** Animal Breeding and Genomics Centre, Wageningen UR Livestock Research, 6700 AH Wageningen, The Netherlands; Animal Breeding and Genomics Centre, Wageningen University, 6700 AH Wageningen, The Netherlands; Department of Environment and Primary Industries, Agribio, La Trobe University, Bundoora, Victoria 3083 Australia; Faculty of Land and Environment, University of Melbourne, Parkville, Victoria 3010 Australia; Dairy Futures Cooperative Research Centre, La Trobe University, Bundoora, Victoria 3083 Australia; La Trobe University, Bundoora, Victoria 3083 Australia

## Abstract

**Background:**

Although simulation studies show that combining multiple breeds in one reference population increases accuracy of genomic prediction, this is not always confirmed in empirical studies. This discrepancy might be due to the assumptions on quantitative trait loci (QTL) properties applied in simulation studies, including number of QTL, spectrum of QTL allele frequencies across breeds, and distribution of allele substitution effects. We investigated the effects of QTL properties and of including a random across- and within-breed animal effect in a genomic best linear unbiased prediction (GBLUP) model on accuracy of multi-breed genomic prediction using genotypes of Holstein-Friesian and Jersey cows.

**Methods:**

Genotypes of three classes of variants obtained from whole-genome sequence data, with moderately low, very low or extremely low average minor allele frequencies (MAF), were imputed in 3000 Holstein-Friesian and 3000 Jersey cows that had real high-density genotypes. Phenotypes of traits controlled by QTL with different properties were simulated by sampling 100 or 1000 QTL from one class of variants and their allele substitution effects either randomly from a gamma distribution, or computed such that each QTL explained the same variance, i.e. rare alleles had a large effect. Genomic breeding values for 1000 selection candidates per breed were estimated using GBLUP modelsincluding a random across- and a within-breed animal effect.

**Results:**

For all three classes of QTL allele frequency spectra, accuracies of genomic prediction were not affected by the addition of 2000 individuals of the other breed to a reference population of the same breed as the selection candidates. Accuracies of both single- and multi-breed genomic prediction decreased as MAF of QTL decreased, especially when rare alleles had a large effect. Accuracies of genomic prediction were similar for the models with and without a random within-breed animal effect, probably because of insufficient power to separate across- and within-breed animal effects.

**Conclusions:**

Accuracy of both single- and multi-breed genomic prediction depends on the properties of the QTL that underlie the trait. As QTL MAF decreased, accuracy decreased, especially when rare alleles had a large effect. This demonstrates that QTL properties are key parameters that determine the accuracy of genomic prediction.

**Electronic supplementary material:**

The online version of this article (doi:10.1186/s12711-015-0124-6) contains supplementary material, which is available to authorized users.

## Background

In livestock breeding programs, genomic information is widely used to estimate genomic breeding values for selection candidates. Genomic estimated breeding values (GEBV) are calculated from marker effects estimated in a reference population that consists of animals with phenotypes and marker genotypes. Accuracy of GEBV for selection candidates, that typically have no phenotypes of their own, depends on the size of the reference population i.e. the larger the size of the reference population, the more accurately breeding values can be predicted, e.g. [1-3]. For numerically small breeds, assembling such a large reference population is challenging, therefore, an attractive approach would be to combine purebred reference populations from different breeds or lines to establish large reference populations [4-7]. However, the benefit of adding another breed or line to the reference population may be reduced by the inconsistency in allele substitution effects across breeds [8-10], by between-breed differences in linkage disequilibrium (LD) between single nucleotide polymorphisms (SNPs) and quantitative trait loci (QTL) that influence a trait across breeds or lines, e.g. [7,11,12], as well as by the absence of close family relationships between breeds or lines [13]. In addition, the accuracy of prediction using both single-breed and multi-breed reference populations may be affected by the properties of the QTL that control a trait, i.e. number of QTL for the trait, joint distribution of QTL allele frequencies across breeds, and distribution of QTL effects.

In *Bos taurus* cattle populations, LD phase is conserved across breeds among SNP alleles at short distances (5 to 30 kb) [11]. Therefore, a high marker density might overcome the problem of differences in LD between SNPs and QTL across breeds or lines [11]. Indeed, simulation studies using high-density markers showed that prediction accuracy increased when reference populations were combined across breeds compared to single-breed reference populations [6,14]. However, in empirical studies, the increase in prediction accuracy was smaller and sometimes absent [12,15,16], even when more than 600 000 SNPs were used [4,17,18]. Part of this difference between accuracies obtained from simulation and empirical studies could be explained by the assumptions made in simulation studies on the properties of the QTL that underlie a trait, which may not completely reflect the reality. One of these QTL properties that could affect prediction accuracy is the pattern of QTL allele frequencies. For most complex traits, the QTL that underlie a trait have a low minor allele frequency (MAF) [19-21]. Due to ascertainment bias of SNP chips [22], SNPs tend to have higher MAF than QTL, which reduces the LD between QTL and SNPs and therefore the accuracy of genomic prediction, particularly across breeds and lines. Besides differences in allele frequencies between SNPs and QTL, differences in allele frequencies of QTL across breeds may also influence prediction accuracy. In extreme cases, QTL may even only segregate in one of the breeds. When the SNPs that flank a breed-specific QTL are segregating across breeds, the apparent effect of SNPs may vary across breeds. The above examples show that the properties of QTL that underlie a trait are likely to affect the accuracy of multi-breed or line genomic prediction.

In spite of potential differences in QTL properties across breeds, most studies on multi-breed genomic prediction estimate only one effect for each SNP across all breeds, e.g. [4,15,23]. Makgahlela *et al.* [24] and Olson *et al.* [25] accounted for differences in SNP effects across breeds by fitting a multi-trait model in which the same trait in different breeds was treated as a different trait and both studies showed a minor increase in prediction accuracy using ~40 000 SNPs. Another way to account for breed-specific SNP effects and at the same time benefit from increasing the size of the reference population by adding another breed could be to estimate an across-breed SNP effect and a within-breed SNP effect. Khansefid *et al.* [26] showed that this can be done by including a random across-breed animal effect and a within-breed animal effect in a genomic best linear unbiased prediction (GBLUP) model.

The first objective of this study was to investigate the effect of the properties of the QTL that underlie the trait on the accuracy of multi-breed genomic prediction. The second objective was to investigate the effect of a GBLUP model with a random across-breed animal and a within-breed animal effect on the accuracy of multi-breed genomic prediction. In this study, real genotypes of Holstein-Friesian and Jersey dairy cows were used. Phenotypes were simulated using different properties of QTL by sampling 100 or 1000 QTL from three different classes of markers with average MAF that ranged from moderately low (representing allele frequencies expected under a neutral model) to extremely low values, and by simulating allele substitution effects using two different models.

## Methods

For this study, two different datasets were used. For the first dataset, including genotypes of Australian cows, samples were collected for DNA extraction as approved by the Department of Primary Industries Victoria Animals Ethics Committee (protocol: 2010-19). For the second dataset, sequence information from the 1000 bull genomes project was used, for which DNA for most animals was extracted from semen. Only for Angus animals, samples were collected for DNA extraction as approved by the New South Wales Department of Primary Industries Animals Ethics Committee.

### Genotypes

Genotypes were available for 3000 Holstein-Friesian cows and 3000 Jersey cows from Australia. Individuals were genotyped with the Illumina BovineHD Beadchip (777 k, Illumina, San Diego, CA) or the Illumina BovineSNP50 Beadchip (50 k, Illumina, San Diego, CA). Animals genotyped at the lower density (50 k) were imputed to high density (777 k) using the software package Beagle 3.0 [27] and a reference population of 1072 animals (Holstein-Friesian and Jersey) that were genotyped with the high-density (777 k) chip. Quality was checked using a larger dataset that included those 6000 individuals. SNPs of low quality based on the same criteria as described in Erbe *et al.* [4] were removed, leaving 606 384 SNPs for the analyses.

In order to obtain plausible QTL allele frequencies that ranged from frequencies of loci that are effectively neutral to frequencies of loci that are expected to have large pleiotropic effects on fitness, sequence data of variants in annotated classes from the 1000 bull genomes project [28] was used. This included sequence information of 129 Holstein-Friesian, 15 Jersey, 47 Angus and 43 Simmental animals. Variants in this dataset were annotated as either synonymous mutations (80 515 mutations), missense mutations (97 296 mutations), and premature stop codon mutations (4064 mutations), with about the same number of variants in each class as presented in Daetwyler *et al.* [28]. More information about the samples, alignment, variant calling and filtering, and annotation of the sequenced animal genomes is in Daetwyler *et al.* [28].

Our aim was to simulate different groups of QTL that had decreasing MAF and that were increasingly more difficult to tag with SNPs on the SNP chip and were equally distributed across the whole genome. Therefore, the three classes of annotated variants that varied in average MAF (Table 1) and MAF pattern [See Additional file 1 Figure S1 and Additional file 2 Figure S2], were used to represent different patterns of QTL MAF; the synonymous mutations represented QTL with on average a moderately low MAF (average MAF of 0.122), the missense mutations represented QTL with on average a very low MAF (average MAF of 0.077), and the premature stop codon mutations represented QTL with on average an extremely low MAF (average MAF of 0.016). It should be noted that these classes of variants were only used to represent differences in patterns of QTL MAF and not differences in biological functions of the QTL.

**Table 1:** **Characteristics of different classes of variants used to simulate QTL**

**Characteristic per class**	**Holstein-Friesian**	**Jersey**	**Total**
**Moderately low average MAF** ^**1,2**^			
Segregating variants	63 119	55 363	65 920
Number of breed-specific variants	10 557	2801	13 358
Percentage of breed-specific variants	16.0	4.2	20.3
Average MAF^1^ of the 65 920 segregating variants (± standard deviation)	0.130 ± 0.169	0.115 ± 0.168	0.122 ± 0.146
**Very low average MAF** ^**1,3**^			
Segregating variants	61 302	49 473	67 097
Number of breed-specific variants	17 624	5795	23 419
Percentage of breed-specific variants	26.3	8.6	34.9
Average MAF^1^ of the 67 097 segregating variants (± standard deviation)	0.082 ± 0.146	0.072 ± 0.142	0.077 ± 0.127
**Extremely low average MAF** ^**1,4**^			
Segregating variants	1804	1245	2142
Number of breed-specific variants	897	338	1235
Percentage of breed-specific variants	41.9	15.8	57.7
Average MAF^1^ of the 2142 segregating variants (± standard deviation)	0.017 ± 0.067	0.015 ± 0.066	0.016 ± 0.059

^1^MAF = minor allele frequency; ^2^annotated as synonymous mutations; ^3^annotated as missense mutations; ^4^annotated as premature stop codon mutations.

Genotypes for the three classes of variants were imputed in 3000 Holstein-Friesian and 3000 Jersey animals with real high-density SNP genotypes [27]. Imputation was done using all sequenced animals from the reference population, which included the Angus and Simmental animals, since it has been shown that using animals from other breeds improves imputation accuracy [28,29]. Allele frequency patterns of the imputed variants were similar to the allele frequency patterns in sequenced animals [See Additional file 1 Figure S1 and Additional file 2 Figure S2]. Other characteristics of the three classes of imputed variants are in Table 1. For imputed and real sequence data, the number of segregating variants was much smaller for the Jersey population than for the Holstein-Friesian population. This is probably due to the small number of Jersey sequenced genomes in the dataset, since more polymorphic SNPs are detected when the group of genotyped individuals is larger [30-32]. Reliabilities (i.e. *R*^*2*^ values) of imputation were low (average reliabilities estimated by Beagle were equal to 0.67 for variants with on average a moderately low MAF, 0.51 for variants with on average a very low MAF, and 0.32 for variants with on average an extremely low MAF), which probably results from the relatively small number of animals with sequence data in combination with the low MAF of the variants. This decrease in reliabilities of imputation as average MAF of variants decreases confirms the assumption that LD between variants with a low MAF and neighboring SNPs on the commercial SNP chip decreases, i.e. that tagging the variants with SNPs on the chip was increasingly more difficult.

### Simulation of phenotypes

Traits that were controlled by QTL with different properties were simulated by varying: (1) the average MAF of the QTL that underlie the trait, by sampling QTL from one of the three classes described above, (2) the number of QTL that underlie the trait, and (3) the distribution of allele substitution effects. In each simulation, 100 or 1000 QTL were sampled assuming that they followed one of the three QTL MAF patterns i.e. moderately low average MAF, very low average MAF, or extremely low average MAF. All variants that segregated in the entire dataset, consisting of 3000 Holstein-Friesian and 3000 Jersey individuals, were considered as potential QTL, which resulted in 65 920 potential QTL with a moderately low average MAF, 67 097 with a very low average MAF, and 2142 with an extremely low average MAF. It should be noted that the percentage of breed-specific variants increased as the MAF of the variants decreased (Table 1).

Allele substitution effects were sampled using two different models: (1) a pseudo-infinitesimal model, where small allele substitution effects were randomly assigned to QTL independently of allele frequency (RANDOM model), and (2) a ‘rare allele, large effect’ model, where larger allele substitution effects were assigned to QTL with a lower MAF such that each QTL explained an equal amount of the total genetic variance (VAR model). Under the RANDOM model, allele substitution effects were randomly sampled from a gamma distribution with a shape parameter of 0.4 and a scale parameter of 1.66, following Meuwissen *et al.* [1]. Under the VAR second model, the variance explained by each QTL was kept constant across all QTL by computing allele substitution effects as $a=\sqrt{\frac{Var(QTL)}{2p\left(1-p\right)}},$ where *a* is the allele substitution effect assuming a purely additive model, *Var*(*QTL*) is the variance of the QTL which is constant across the QTL and was set to 1, and *p* is the allele frequency of the QTL across all 6000 individuals (3000 Holstein-Friesian and 3000 Jersey cows). Under the two models, both alleles at a given QTL had an equal chance to have a positive or a negative effect on the simulated trait and the effect was the same in both breeds. The simulated allele substitution effects were multiplied by the genotype codes (0, 1, or 2) to calculate a true breeding value (TBV) for each individual. Over all individuals and across the breeds, TBV were rescaled to a mean of 0 and a variance of 1.

Allele frequencies for the loci selected as QTL differed between the two breeds [See Additional file 3 Figure S3]). These differences in allele frequencies resulted in differences in average TBV between breeds. To calculate the genetic variance as the variance across TBV, breed effects were first subtracted from all TBV to avoid breed effects influencing the simulated heritability. Thereafter, the environmental effect per individual was sampled from a normal distribution with a mean of 0 and variance $\left(\frac{1}{h^2}-1\right)$*(variance of TBV corrected for breed effect). For each individual, the phenotype was calculated as the sum of its TBV, including its breed effect and the randomly sampled environmental effect.

In this study, a rather simple situation was simulated to be able to investigate the effect of QTL properties on the accuracy of both single- and multi-breed genomic prediction. Heritabilities and allele substitution effects were assumed to be the same across breeds, such that phenotypic differences between breeds were only due to differences in QTL allele frequencies. Phenotypes were simulated using a heritability of 0.8, which is similar to the heritability of daughter yield deviation of a bull for milk yield if the bull has approximately 100 daughters. We chose this rather high heritability value to achieve high accuracies of genomic prediction, which resulted in more pronounced differences in accuracies between the different scenarios for the small reference population size used in the simulations. According to the formula of Daetwyler *et al.* [2,33], a trait with a heritability of 0.8 is expected to yield the same accuracy as a trait with a heritability of 0.25 but using a reference population that includes 3.2 times more animals.

To decide on the number of replicates, the variance of the squared accuracy (*r*^2^) was calculated from the sampling variance of a correlation coefficient as [34]:

$$ Var\left({r}^2\right)=\frac{{\left(1-{r}^2\right)}^2}{N-1}, $$

where *N* is the number of selection candidates. Thereafter, the required number of replicates (*n*) was calculated as [35]:

$$ n>\frac{\left({1.96}^2*Var\left({r}^2\right)\right)}{0.02^2}, $$

where 1.96 refers to the z-value on the standard normal distribution relating to a confidence interval of 95%, and 0.02 is the maximum allowable difference between the estimated and true mean. This resulted in a maximum required number of replicates of 9.62 with an actual accuracy of 0, and a minimum required number of replicates of 0.004 with an actual accuracy of −0.99 or 0.99. Thus, 10 replicates are sufficient to cover the whole spectrum of possible accuracies.

### Investigating the accuracy of genomic prediction

For each replicate, the accuracy of genomic prediction was empirically calculated for a fixed group of 1000 Holstein-Friesian and 1000 Jersey selection candidates that were selected from the 3000 animals per breed that were used in this study. Due to the presence of overlapping generations and the use of cow data with small progeny groups, selection candidates were randomly sampled from the full dataset. The other 2000 Holstein-Friesian and 2000 Jersey cows were used as reference animals in seven reference populations (Table 2), with different numbers of Holstein-Friesian and Jersey individuals that ranged from a single-breed reference population to a multi-breed reference population with equal numbers of animals of both breeds. Each of the smaller reference populations was a random subset from the larger reference populations.

**Table 2:** **Overview of the different reference populations**

	**Reference population**	
**Scenarios**	**Number of Holstein-Friesian**	**Number of Jersey**
**1**	2000	2000
**2**	2000	500
**3**	2000	100
**4**	2000	0
**5**	500	2000
**6**	100	2000
**7**	0	2000

Since LD patterns between QTL and SNPs differed across breeds and some QTL segregated only in one of the breeds, SNP effects were expected to differ across breeds. To account for these differences in SNP effects, a Genomic-Relatedness-Matrix Residual Maximum Likelihood model (GREML) including both a random across-breed animal effect and a within-breed animal effect was run in ASReml [36]. A GREML model has the same features as the commonly known GBLUP model (assuming a normal distribution of SNP effects), but it estimates the variances and the breeding values simultaneously using REML. This was done using the following model, hereafter called the base model:

$$ \mathbf{y}={\mathbf{1}}_n\mu +\mathbf{Z}{\mathbf{g}}_a+\mathbf{Z}{\mathbf{g}}_w+\mathbf{e}, $$

where **y** is a vector containing the simulated phenotypes, **1**_*n*_ is a vector of ones, *μ* is the overall mean across breeds, **g**_*a*_ and **g**_*w*_ are vectors of the genomic breeding values predicted either across breeds or within breeds (**g**_*a*_ ~ $N\left(0,{\mathbf{G}}_a{\upsigma}_{g_a}^2\right)$ and **g**_*w*_ ~ $N\left(0,{\mathbf{G}}_w{\upsigma}_{g_w}^2\right)$), **Z** is an incidence matrix that allocates genomic breeding values (both **g**_*a*_ and **g**_*w*_) to the individuals and **e** is a vector containing the residuals ~ $N\left(0,\mathbf{I}{\upsigma}_e^2\right)$. Note that only one ${\upsigma}_{g_a}^2$ and one ${\upsigma}_{g_w}^2$ were estimated, which reflect the variances in the base population of the genomic relationship matrices (**G**_*a*_ and **G**_*w*_), which was set to be the population immediately before Holstein-Friesian and Jersey breeds diverged by using the method of Erbe *et al.* [4]. As a first step to calculate **G**_*a*_ and **G**_*w*_, the **G** matrix was calculated as [4]:

$$ \mathbf{G}=\frac{\mathbf{WW}\hbox{'}}{2{\displaystyle \sum_{j=1}^n{p}_j\left(1-{p}_j\right)}}, $$

where *n* is the number of loci, **W** is a matrix of standardized genotypes for individual *i* at locus *j* calculated as *w*_*ij*_ = *g*_*ij*_ − 2*p*_*j*_, where *g*_*ij*_ codes the genotype as 0, 1 and 2, and *p*_*j*_ is the allele frequency for the second allele (for which the homozygote genotype is coded 2) calculated as *p*_*j*_ = *αp*_*j*,*HF*_ + (1 − *α*)*p*_*j*,*Jer*_. In this last equation, *p*_*j*,*HF*_ is the allele frequency in the Holstein-Friesian population, *p*_*j*,*Jer*_ is the allele frequency in the Jersey population and *α* is calculated as $\alpha =\frac{F_{Jer}}{F_{Jer}+{F}_{HF}}$, and represents the proportion of Holstein-Friesian haplotypes in the ancestral population. The inbreeding coefficient for the Jersey population was calculated as:

$$ {F}_{Jer}=1-\frac{{\displaystyle \sum_{j=1}^n2{p}_{j,Jer}\left(1-{p}_{j,Jer}\right)}}{{\displaystyle \sum_{j=1}^n\left[{p}_{j,HF}\left(1-{p}_{j,Jer}\right)+{p}_{j,Jer}\left(1-{p}_{j,HF}\right)\right]}}. $$

The inbreeding coefficient for the Holstein-Friesian population was calculated in the same way by substituting the two breeds accordingly. As described by Erbe *et al.* [4], inbreeding in **G** can be adjusted for the inbreeding that occurred relative to the base set at the time of divergence of the two breeds as G* = **G**(1 − *F*) + 2*F*. In this equation, *F* is the inbreeding relative to an F1 base population calculated as $F=\frac{F_{Jer}{F}_{HF}}{F_{Jer}+{F}_{HF}}$. The relationship matrix based on the pedigree, **A**, was rescaled to the same base by rescaling the within-Holstein-Friesian block as A* = **A**[1 − (*F*_*HF*_ − *f*_*HF*_)] + 2(*F*_*HF*_ − *f*_*HF*_), in which *f*_*HF*_ is the amount of inbreeding in the Holstein-Friesian population since the base of the pedigree. The within-Jersey block was rescaled in the same way and the across-breed block was set to 0. Thereafter, the rescaled G* matrix was regressed back to the rescaled A* matrix following Yang *et al.* [21] and Goddard *et al.* [37] to calculate **G**_*a*_. The regression was done separately across and within breed as well as per bin of pedigree relationship (<0.10, 0.10-0.25, 0.25-0.50, > 0.5), because the sampling error on elements of G* depends on the level of family relationships. Across these bins of relationships, the different regression coefficients ranged from 0.994 to 0.999 when all 606 384 SNPs were used to calculate **G**_*a*_. The **G**_*w*_ matrix was formed from the **G**_*a*_ matrix by setting the elements between individuals of different breeds to zero, while the within-breed elements of **G**_*w*_ were equal to the corresponding elements in **G**_*a*_.

In this base model, genomic breeding values were predicted across breeds as well as within breeds. For each selection candidate, the genomic breeding values across and within breed were summed to calculate the total genomic breeding value. The accuracy of genomic prediction was calculated per breed as the correlation between the total genomic breeding values and the simulated true breeding values of all selection candidates of that breed.

Analyses were performed using different numbers of SNPs to set-up **G**_*a*_ and **G**_*w*_, namely: (1) 606 384 SNPs, (2) 60 000 SNPs, (3) 606 384 SNPs plus the genotypes of all imputed variants representing QTL, and (4) 60 000 SNPs plus the genotypes of all imputed variants representing QTL. The 60 000 SNPs were randomly selected from the 606 384 SNPs to study the accuracy that could be achieved with a lower marker density. When genotypes for the imputed variants representing QTL were included in the dataset used to calculate **G**_*a*_ and **G**_*w*_, genotypes of all imputed variants in the three classes were used i.e. 80 515 variants with a moderately low average MAF, 97 296 with a very low average MAF and 4064 with an extremely low average MAF. In this way, the potential accuracy of genomic prediction was studied when the causal mutations, i.e. the QTL, were included in the marker dataset.

The power of the base model to separate across- and within-breed animal effects was investigated for one of the scenarios, namely the RANDOM scenario with 1000 QTL and 2000 Holstein-Friesian and 2000 Jersey animals in the reference population. Due to computational reasons, only one of the scenarios was investigated. The base model that included a random across-breed animal effect and a within-breed animal effect, was run once for each specific replicate in this scenario and the total genetic variance was calculated. Thereafter, the model was run again by fixing the within-breed variance to 1, 10, 20, 30, 40, 50, 60, 70, 80, 90, 99 % of the total genetic variance and assigning the remaining part to the across-breed variance. To test for significance, twice the difference in log-likelihood between the model with fixed variance components and the model with estimated variance components was compared with the 5% significance threshold (2.71) taken from a mixed Chi-square distribution with 0 and 1 degrees of freedom.

To investigate the advantage in terms of prediction accuracy of using a GBLUP type of model with a random across-breed animal effect and a within-breed animal effect over a model with only a random across-breed animal effect, the analyses were repeated using a model where **Zg**_*w*_ was removed. The effect of a fixed breed effect on accuracy of multi-breed genomic prediction was also studied by running the base model including breed as a fixed effect. Both alternative models were run for the RANDOM and VAR scenarios using all reference populations when 100 QTL controlled the trait.

## Results

The results presented in this section are the averages across the 10 replicates, with standard errors computed across the replicates. In general, the standard errors across replicates were small. To further investigate if 10 replicates were sufficient for this study, the impact of the number of replicates was analyzed by comparing the averages after 10 replicates with the averages after the first five replicates. In general, the absolute difference in accuracy was only ~0.01 between the averages after five and 10 replicates for all scenarios using the base model and QTL with a moderately low average MAF, very low average MAF or extremely low average MAF. Standard errors were, as expected, slightly higher with five replicates. The low standard errors and the small differences in averages after five and 10 replicates indicate that using only 10 replicates did not affect the conclusions of our study.

### QTL properties

**Figure 1:**
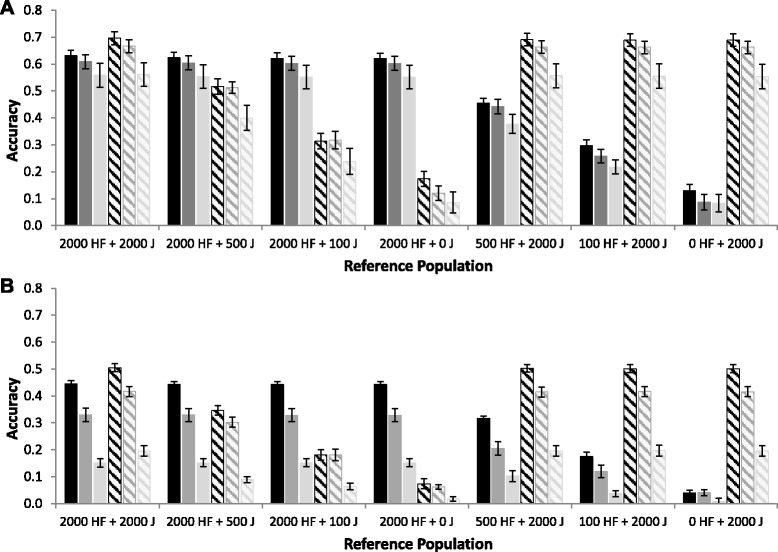
Accuracies of genomic prediction for traits that are controlled by QTL with different properties when 100 QTL underlie the trait. Average accuracies of genomic prediction (± standard errors) for Holstein-Friesian (HF, solid fill) and Jersey (J, diagonal fill) animals using a model that included a random across-breed animal effect and a within-breed animal effect, 606 384 SNPs, seven different reference populations and using simulated allele substitution effects **(A)** randomly sampled from a gamma distribution or **(B)** with each QTL explaining an equal proportion of the genetic variance, when 100 QTL underlying the trait were sampled from variants with on average a moderately low allele frequency (black), very low minor allele frequency (dark grey) or extremely low minor allele frequency (light grey).

Average accuracies for the base model using all 606 384 SNPs for the different reference populations are in Figure 1 when 100 QTL controlled the simulated trait, both for the RANDOM (A) and VAR (B) scenarios. For all reference populations, accuracies were greater for the RANDOM scenario than for the VAR scenario, regardless of the average MAF of QTL. Moreover, accuracies were slightly greater for Jersey selection candidates than for Holstein-Friesian selection candidates when the number of individuals in the reference population from the evaluated breed was the same, which reflects the smaller effective population size of this breed.

As the number of reference individuals of a breed decreased, the achieved prediction accuracies for the selection candidates from the same breed decreased as expected for all scenarios. For the RANDOM scenario, prediction accuracy decreased by ~0.51 for the Jersey and ~0.01 for the Holstein-Friesian selection candidates when the number of Jersey individuals changed from 2000 to 0 in the reference population, and it decreased by ~0.01 for the Jersey and ~0.50 for the Holstein-Friesian selection candidates when the number of Holstein-Friesian individuals changed from 2000 to 0 in the reference population. For the VAR scenario, the decrease in accuracy due to a decreasing number of animals from the breed itself was also large, although this decrease was less pronounced due to smaller accuracies, and the decrease in accuracy due a decreasing number of animals from the other breed was negligible. Thus, the effect of including another breed in the reference population on prediction accuracy was small for both scenarios.

In general, accuracies were greatest for QTL with a moderately low average MAF and smallest for QTL with an extremely low average MAF. The differences in accuracies between classes of QTL with different average MAF were more pronounced for the VAR scenario than for the RANDOM scenario, mainly as a result of a smaller accuracy for QTL with a very low average MAF and a much smaller accuracy for QTL with an extremely low average MAF. These results are consistent with the estimated heritabilities for each scenario (Table 3); estimated heritabilities decreased when the average MAF of QTL decreased and the differences were more pronounced for the VAR scenario than for the RANDOM scenario. For all scenarios, the estimated heritability was below the simulated heritability, but for the RANDOM scenario, the differences were small. This indicates that it was difficult for the GBLUP model to capture all the genetic variance when the QTL that underlie the simulated trait had on average a low MAF, especially when rare alleles had a large effect.

**Table 3:** **Average estimated heritabilities of QTL with different properties**

**Sc.**	**Nb HF** ^**1**^	**Nb J** ^**2**^	**RANDOM**			**VAR**		
			**Moderately low MAF** ^**3**^	**Very low MAF** ^**3**^	**Extremely low MAF** ^**3**^	**Moderately low MAF** ^**3**^	**Very low MAF** ^**3**^	**Extremely low MAF** ^**3**^
**1**	2000	2000	0.78 (0.003)	0.77 (0.002)	0.72 (0.011)	0.60 (0.001)	0.44 (0.002)	0.21 (0.002)
**2**	2000	500	0.76 (0.004)	0.75 (0.006)	0.70 (0.023)	0.54 (0.002)	0.38 (0.004)	0.18 (0.001)
**3**	2000	100	0.75 (0.005)	0.75 (0.007)	0.70 (0.027)	0.54 (0.002)	0.36 (0.004)	0.18 (0.002)
**4**	2000	0	0.75 (0.005)	0.75 (0.007)	0.70 (0.029)	0.54 (0.002)	0.37 (0.004)	0.18 (0.002)
**5**	500	2000	0.79 (0.004)	0.78 (0.002)	0.70 (0.008)	0.64 (0.001)	0.47 (0.002)	0.22 (0.006)
**6**	100	2000	0.78 (0.007)	0.76 (0.005)	0.62 (0.017)	0.62 (0.001)	0.44 (0.002)	0.19 (0.004)
**7**	0	2000	0.78 (0.008)	0.76 (0.006)	0.58 (0.025)	0.61 (0.002)	0.42 (0.002)	0.17 (0.004)

Average heritabilities (standard errors across replicates) estimated with a model including a random across-breed animal effect and a within-breed animal effect and using 606 384 SNPs to calculate the genomic relationship matrix using different reference populations, different average minor allele frequencies (MAF) of the 100 QTL that underlie the trait and using simulated allele substitution effects randomly sampled from a gamma distribution (RANDOM) or with each QTL explaining an equal proportion of the genetic variance (VAR).

Sc. = scenarios; ^1^Nb HF = number of Holstein-Friesian animals; ^2^Nb J = number of Jersey animals; ^3^MAF = minor allele frequency.

**Figure 2:**
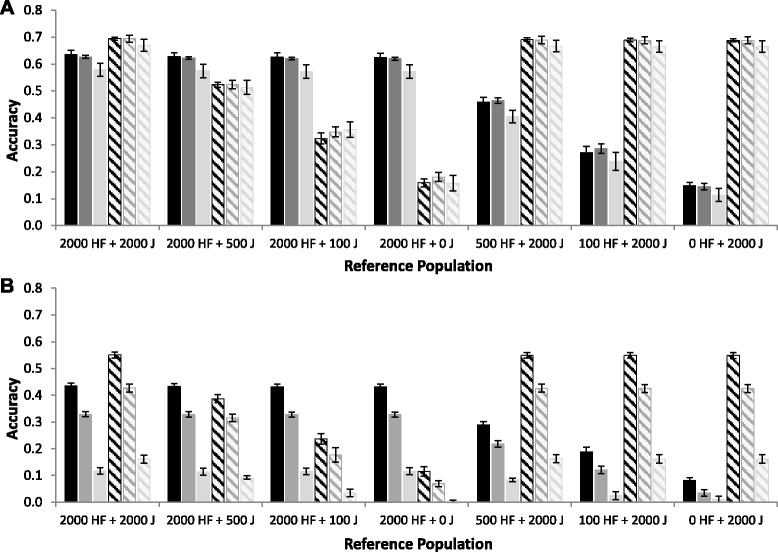
Accuracies of genomic prediction for traits that are controlled by QTL with different properties when 1000 QTL underlie the trait. Average accuracies of genomic prediction (± standard errors) for Holstein-Friesian (HF, solid fill) and Jersey (J, diagonal fill) animals using a model that included a random across-breed animal effect and a within-breed animal effect, 606 384 SNPs, seven different reference populations and using simulated allele substitution effects **(A)** randomly sampled from a gamma distribution or **(B)** with each QTL explaining an equal proportion of the genetic variance, when 1000 QTL underlying the trait were sampled from variants with on average a moderately low allele frequency (black), very low minor allele frequency (dark grey) or extremely low minor allele frequency (light grey).

For the RANDOM scenario, the number of QTL underlying a trait had a limited effect on prediction accuracies (Figures 1 and 2); accuracies were slightly greater for QTL with a very low average MAF (~0.03) or extremely low average MAF (~0.07) when 1000 QTL instead of 100 controlled the trait. This reduced the effect of the average MAF of QTL on accuracy with 1000 QTL compared to 100 QTL. For the VAR scenario, the effect of the number of QTL on accuracy was very small for all situations (Figures 1 and 2). Estimated heritabilities with 1000 QTL underlying the trait were similar to those with 100 QTL underlying the trait, both for the RANDOM and VAR scenarios [See Additional File 4 Table S1].

### Marker densities and mutations

**Figure 3:**
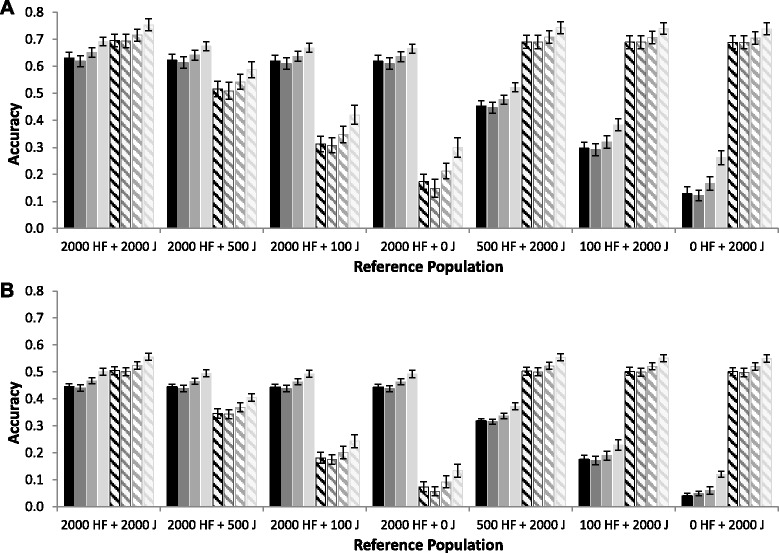
Accuracies of genomic prediction using different marker densities to calculate the genomic relationship matrix. Average accuracies of genomic prediction (± standard errors) for Holstein-Friesian (HF, solid fill) and Jersey (J, diagonal fill) animals using a model that included a random across-breed animal effect and a within-breed animal effect, seven different reference populations and using simulated allele substitution effects **(A)** randomly sampled from a gamma distribution or **(B)** with each QTL explaining an equal proportion of the genetic variance, when 100 QTL underlying the trait were sampled from variants with on average a moderately low minor allele frequency. The genomic relationship matrices were calculated using 606 384 SNPs (black), 60 000 SNPs (dark grey), 606 384 SNPs plus all sampled QTL (grey), or 60 000 SNPs plus all sampled QTL (light grey).

With 100 QTL underlying the trait, average accuracies achieved with the base model that used genomic relationship matrices based on different marker densities, with or without the simulated QTL, are in Figure 3 for the RANDOM scenario (A) and VAR scenario (B). For both scenarios, a decrease in the number of SNPs used to calculate the genomic relationship matrices from 606 384 to 60 000 resulted in similar accuracies of genomic prediction, although values were slightly, but consistently, lower (~0.007) with 60 000 SNPs than with 606 384 SNPs. Estimated heritabilities using 60 000 or 606 384 SNPs were also similar (Table 4).

**Table 4:** **Average estimated heritabilities using different marker densities to calculate the genomic relationship matrix**

**Sc.**	**Nb HF** ^**1**^	**Nb J** ^**2**^	**RANDOM**				**VAR**			
			**600 k**	**60 k**	**600 k + QTL**	**60 k + QTL**	**600 k**	**60 k**	**600 k + QTL**	**60 k + QTL**
**1**	2000	2000	0.78 (0.003)	0.77 (0.003)	0.80 (0.002)	0.84 (0.001)	0.59 (0.001)	0.58 (0.001)	0.61 (0.001)	0.64 (0.001)
**2**	2000	500	0.76 (0.004)	0.74 (0.004)	0.78 (0.003)	0.82 (0.003)	0.54 (0.003)	0.53 (0.003)	0.57 (0.003)	0.59 (0.003)
**3**	2000	100	0.75 (0.005)	0.73 (0.005)	0.77 (0.004)	0.80 (0.005)	0.54 (0.004)	0.53 (0.004)	0.57 (0.004)	0.59 (0.004)
**4**	2000	0	0.75 (0.005)	0.73 (0.005)	0.77 (0.004)	0.81 (0.005)	0.55 (0.004)	0.54 (0.004)	0.58 (0.004)	0.60 (0.004)
**5**	500	2000	0.79 (0.004)	0.78 (0.005)	0.80 (0.003)	0.83 (0.002)	0.61 (0.006)	0.60 (0.005)	0.63 (0.005)	0.66 (0.005)
**6**	100	2000	0.78 (0.007)	0.77 (0.007)	0.80 (0.006)	0.82 (0.005)	0.58 (0.006)	0.58 (0.006)	0.60 (0.006)	0.63 (0.005)
**7**	0	2000	0.78 (0.008)	0.77 (0.008)	0.79 (0.007)	0.82 (0.006)	0.56 (0.006)	0.55 (0.006)	0.57 (0.005)	0.60 (0.005)

Average heritabilities (standard errors across replicates) estimated with a model including a random across-breed animal effect and a within-breed animal effect using different reference populations, 100 QTL underlying the trait with on average a moderately low minor allele frequency and using simulated allele substitution effects randomly sampled from a gamma distribution (RANDOM) or with each QTL explaining an equal proportion of the genetic variance (VAR). The genomic relationship matrix was calculated using 606 384 SNPs (600 k), 60 000 SNPs (60 k), 606 384 SNPs plus all sampled QTL (600 k + QTL), or 60 000 SNPs plus all sampled QTL (60 k + QTL).

Sc. = scenarios; ^1^Nb HF = number of Holstein Friesian animals; ^2^Nb J = number of Jersey animals.

Adding the genotypes of the simulated QTL to the SNPs used to calculate the genomic relationship matrices increased prediction accuracy (Figure 3), and the percentage of increase was higher when the average MAF of QTL was lower [See Additional file 5 Figure S4 and Additional file 6 Figure S5]. This increase in accuracy was greater when 60 000 SNPs were used (increase in accuracy of ~0.08 and ~0.06 for the RANDOM and VAR scenarios, respectively) than when 606 384 SNPs were used (increase in accuracy of ~0.02 for both scenarios). Thus, prediction accuracies were greatest when 60 000 SNPs plus the genotypes of the simulated QTL were used to calculate the genomic relationship matrices. As expected, the same pattern was observed with estimated heritabilities (Table 4). This indicates that including the simulated QTL in the marker set to calculate genomic relationship matrices improved the ability of the model to capture all the genetic variance present in the reference population, probably because the QTL can capture the effects without depending on LD between marker and QTL.

### Different models

The base model of this study contained a random across-breed animal effect and a within-breed animal effect to account for differences in SNP effects across breeds. For the multi-breed reference populations, the proportion of variance explained by the within-breed animal component was equal to ~27% and ~52% for the RANDOM and VAR scenarios, respectively, when QTL had a moderately low average MAF, ~33% and ~53% when QTL had a very low average MAF, and ~40% and ~63% when QTL had an extremely low average MAF.

**Figure 4:**
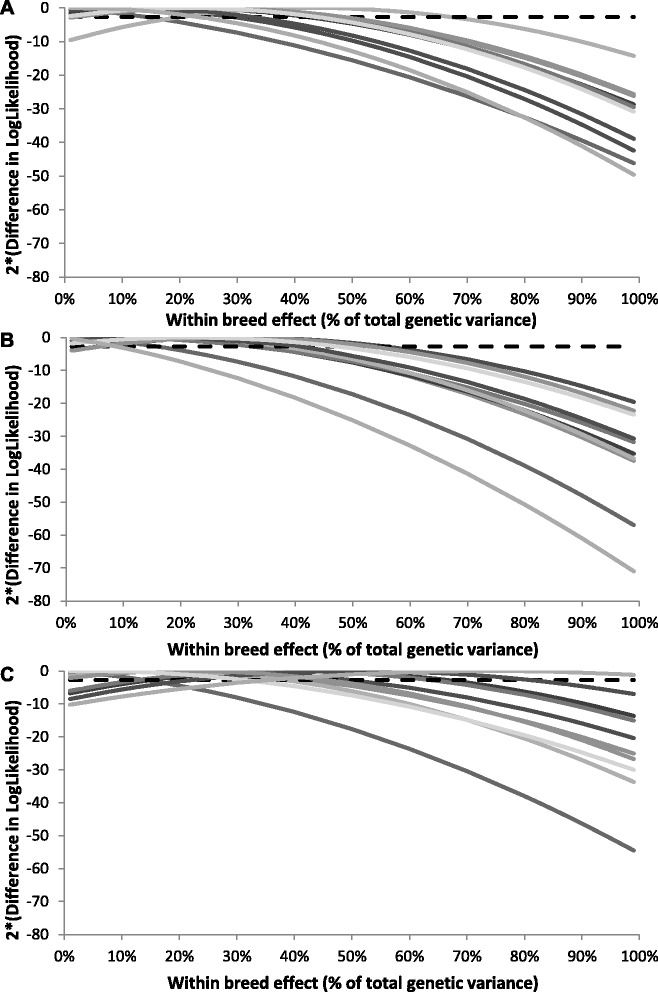
Log likelihood comparison of models with fixed or estimated random across-breed and within-breed animal effects. Twice the difference in log-likelihood for each of the 10 replicates and 5% significance threshold (black dotted line) using models with fixed variance components for the random across-breed animal effect and a within-breed animal effect compared to a model that estimated both variance components. The genomic relationship matrix was calculated based on 606 384 SNPs, the reference population consisted of 2000 Holstein Friesian and 2000 Jersey animals, allele substitution effects were sampled from a gamma distribution, when 1000 QTL underlying the trait were sampled from variants with on average a **(A)** moderately low allele frequency, **(B)** very low minor allele frequency or **(C)** extremely low minor allele frequency.

The power to separate across-breed animal and within-breed animal effects was investigated in Figure 4. This figure shows that for the three classes of QTL with different average MAF and for most of the replicates, the model that estimated across- and within-breed animal variances was not significantly better than a model without a random within-breed animal effect (*P* < 0.05). This is because the log-likelihood is rather flat. Moreover, prediction accuracies and heritabilities estimated with the base model that included a random across-breed animal effect and a within-breed animal effect were very similar to those estimated with a model without a random within-breed animal effect for all scenarios (results not shown). These results indicate that the power to separate across- and within-breed animal effects was limited in our simulated data. Similar prediction accuracies were achieved with a model that included a fixed breed effect (results not shown). Thus, for all scenarios for which a random within-breed animal effect and/or fixed breed effect is included in the model, accuracies of genomic prediction were not affected, and therefore, they are not shown.

## Discussion

### Accuracy of multi-breed genomic prediction

For an accurate prediction of genomic breeding values, a large group of animals with both genotypes and phenotypes is required, e.g. [1-3]. Therefore, an attractive approach is to enlarge small reference populations of a particular breed by using information from other breeds. This might be especially interesting for traits that are difficult to measure, such as feed efficiency and dry matter intake in dairy cattle [38,39], and for numerically small breeds. In this study, the effect of adding another breed to the reference population on prediction accuracy was investigated in different scenarios using Holstein-Friesian and Jersey animals. Accuracy of genomic prediction was not significantly increased by adding 2000 individuals of the other breed to a reference population of animals from the same breed as the selection candidates regardless of marker density. The accuracy of across-breed genomic prediction, i.e. using a reference population consisting only of individuals from the other breed, ranged from 0.01 to 0.19. The positive accuracies of across-breed genomic prediction indicated that useful information was present in the other breed, although adding animals from the other breed to the reference population did not increase prediction accuracy. This suggests that the number of reference individuals from the other breed compared to the number of reference individuals from the breed of the selection candidates was relatively too small to see an increase in accuracy, as suggested by Hozé *et al.* [40]. The benefit of using a multi-breed reference population might also depend on the model used to analyze the data, Bayesian models, for example, might gain more from multiple breeds [41].

### Effect of QTL properties on the accuracy of genomic prediction

The first objective of this study was to investigate the effect of properties of QTL that underlie the trait on the accuracy of multi-breed genomic prediction using Holstein-Friesian and Jersey animals. Phenotypes of traits that are controlled by QTL with different properties were simulated by sampling 100 or 1000 QTL from three different classes of variants that had an average MAF ranging from moderately low to extremely low, and by sampling allele substitution effects either based on a model where effect size was independent of allele frequency (RANDOM) or based on a ‘rare allele, large effect’ model (VAR). The three different classes of variants were imputed using sequenced animal genomes, such that the QTL displayed characteristics that were present on the actual bovine genome. Our results showed that the accuracy of both single-breed and multi-breed genomic prediction was influenced by the properties of the QTL that control the trait. A lower QTL MAF decreased prediction accuracy and this effect was more pronounced when QTL with the lowest MAF had the largest effect, which is consistent with the results from other studies that showed that the prediction model could better capture the genetic variance and provided a greater accuracy of genomic prediction when a small group of QTL explained a large part of the genetic variance [42,43].

A decrease in QTL MAF was expected to decrease accuracy of multi-breed genomic prediction, since the percentage of breed-specific variants increased when the MAF of the variants decreased, thereby reducing the potential benefit of adding another breed. Moreover, LD between SNPs and QTL decreases as the allele frequency of QTL becomes more extreme, due to ascertainment bias of the SNPs on the chip [22]. The existence of ascertainment bias was confirmed by the fact that imputation reliabilities decreased when QTL MAF decreased and that the prediction accuracies increased most when QTL had the lowest MAF and QTL genotypes were added to the markers. Moreover, the low LD between SNPs and QTL is reflected in the increasing underestimation of the heritability as the average QTL MAF decreased. This is in agreement with other studies, that showed that simulating QTL with a low MAF resulted in underestimated heritability estimates [21,44] and lower accuracy of genomic prediction [44,45]. QTL for many complex traits have a low MAF [19-21], which indicates that the probability of underestimating the heritability for those traits is high. Heritability may also be underestimated because only a subset of the animals from a population is used in the analyses. When QTL MAF are low and the size of the reference population is small, the probability that all these QTL are segregating in the reference population is reduced. Therefore, the increase in accuracy of genomic prediction achieved by enlarging the reference population, as shown by e.g. [1-3], might not only result from a more accurate prediction of SNP effects, but also from capturing a larger proportion of the alleles that segregate in the complete population.

**Figure 5:**
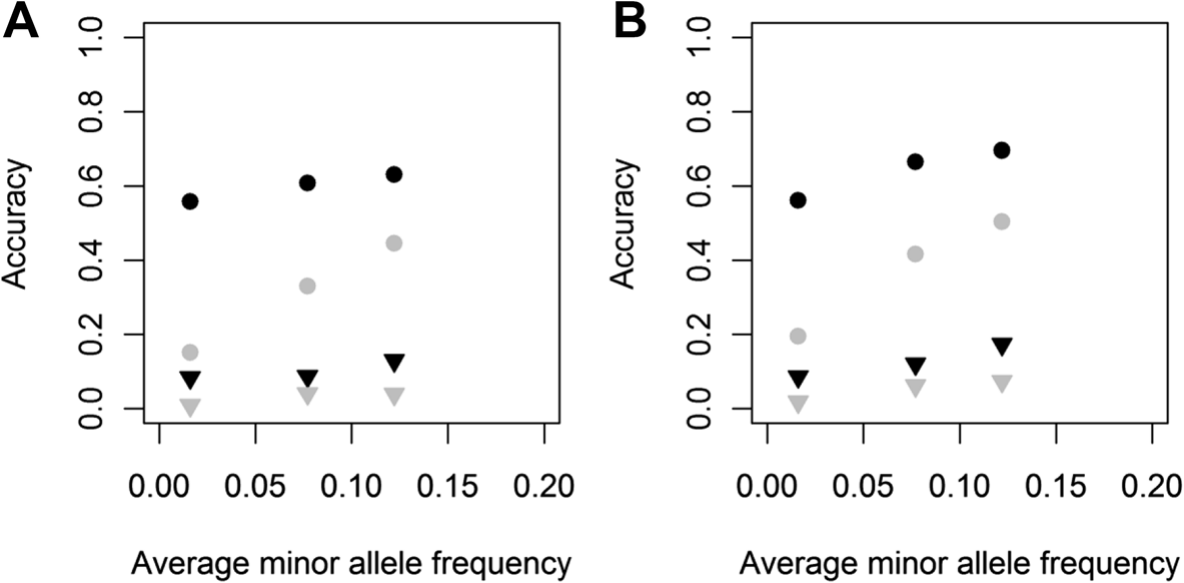
Accuracy of across- and multi-breed genomic prediction versus average minor allele frequency of QTL. The average accuracy of across- and multi-breed genomic prediction for **(A)** Holstein-Friesian and **(B)** Jersey selection candidates versus the average minor allele frequency of the 100 simulated QTL. Black points represent the scenarios with allele substitution effects randomly sampled from a gamma distribution and grey points represent the scenario with each QTL explaining an equal proportion of the genetic variance. The circles represent the accuracy for the multi-breed reference population with 2000 Holstein-Friesian and 2000 Jersey animals, the triangles represent the accuracy of across-breed genomic prediction with a reference population of 2000 animals from the other breed.

Many previous simulation studies have simulated QTL based on SNP characteristics [6,10,14]. However, the SNPs that are commonly used on chips are selected to have a reasonably high MAF and to segregate in multiple breeds. In our data, the average MAF of the SNPs across breeds was 0.27, which is much higher than the average MAF of the other variants (Table 1). As shown in Figure 5, prediction accuracies increase as the average QTL MAF increases; therefore, it is clear that using the pattern of SNP MAF to simulate QTL will result in a substantially larger expected accuracy of both across-breed and multi-breed genomic prediction. This can explain why the benefits of using information from another breed are much larger in other simulation studies compared to our simulation study [6,10,14] and compared to empirical studies, e.g. [4,12,15].

It should be noted that there are two caveats regarding our results, but we consider that they do not affect the overall conclusions greatly. First, the effect of low MAF on accuracy and heritability may be somewhat exaggerated by the imperfect imputation of causal variants. This means that the QTL are not as well tracked by the SNPs as they should be. Second, the formula used to calculate the **G** matrix might be more appropriate for the scenario with allele substitution effects that are sampled independently of allele frequencies than for the scenario using the ‘rare allele, large effect’ model, which might be better analyzed by the **G** matrix described by Yang *et al.* [21]. However, for a fair comparison of the scenarios, we decided to use the same **G** matrix for both scenarios.

### Marker densities and mutations

In this study, the data was analyzed with a GBLUP type of model using genomic relationship matrices based on 606 384 or 60 000 SNPs. Reducing the number of SNPs from 606 384 to 60 000 resulted in similar accuracies of genomic prediction. This is in agreement with empirical studies using dairy cattle data that showed that increasing the number of SNPs from 50 k to high-density (777 k) had almost no effect on the accuracy of multi-breed genomic prediction, e.g. [4,17], in contrast to earlier expectations [11].

For all scenarios, accuracy of genomic prediction was slightly greater when the simulated QTL were added to the subset of markers used to calculate the genomic relationship matrices. This indicates that the model could better capture QTL effects with the markers, which led to higher estimated heritabilities and accuracies, when the simulated QTL were used as markers, which was also shown in other studies [46,47]. The increase in prediction accuracy due to adding the simulated QTL was larger when 60 000 SNPs were used than when 606 384 SNPs were used. This is likely an artifact of the GBLUP model for which all markers are assumed to explain the same amount of variance. This means that as the number of markers increases, each marker effect is *a priori* smaller. Thus, with a larger number of markers, the effects of true markers in the dataset are diluted to a greater degree. By using sequence data in the analyses, the causal variants or QTL are supposed to be included in the data, as well as a large number of other variants. Therefore, on the one hand, the expected benefit of sequence data achieved with a GBLUP model is small, and smaller than that with Bayesian models, which allow some marker effects to be zero, as demonstrated by Meuwissen and Goddard [46]. On the other hand, our result does demonstrate that if the marker set can be enriched with real causative mutations from the sequence data, as we did here by including the QTL in the SNP dataset, accuracies can be increased. The larger increase in prediction accuracy achieved with a smaller number of other variants in the dataset highlights the importance to filter sequence variants that are included in genomic prediction, for example by using biological information [48].

Both in the single-breed and multi-breed scenarios using Holstein-Friesian and Jersey animals, the percentage of increase in accuracies due to adding the QTL genotypes to the markers was higher when the average MAF of QTL was lower. This can be explained by the fact that the QTL with a lower MAF were in lower LD with the SNPs on the chip, particularly across breeds. Besides differences in LD across breeds, the accuracy of multi-breed genomic prediction might also be influenced by other factors, such as the absence of family relationships or differences in allele frequencies, e.g. [2,13,49]. As explained by Daetwyler *et al.* [2], a QTL with a large effect and a low allele frequency in one breed can be imprecisely estimated within that breed. Since that QTL only explains a small proportion of the genetic variance in that breed, the negative effect on the accuracy of single-breed genomic prediction might be small. If the estimated effect was used to predict breeding values for another breed, the effect on accuracy would be more detrimental when the allele frequency of that QTL is higher in that breed. This indicates that it is important that the QTL and SNPs that segregate in the selection candidate population are also segregating with a reasonable allele frequency in the reference population to be able to estimate the effects accurately. When the relationships between selection candidates and reference individuals are larger, the probability that SNPs and QTL segregating in the selection candidate population are segregating in the reference population becomes higher as well. Overall, these results indicate that the accuracy of across-breed genomic prediction is small because of differences in LD, e.g. [7,11], absence of family relationships, e.g. [13,49], and differences in allele frequency across breeds, e.g. [2]; in addition, all these factors are probably entangled with each other.

### Effect of random within-breed animal effect on the accuracy of genomic prediction

The second objective of this study was to investigate the effect of including a random across-breed animal effect and a within-breed animal effect in a GBLUP model on the accuracy of multi-breed genomic prediction. Our results showed that, in contrast to our expectations, adding a random within-breed animal effect did not influence prediction accuracy. In particular, if the QTL were breed-specific and if the SNPs segregated in both breeds, which was to a high extent the case when the average MAF of QTL was extremely low, an increase in accuracy due to the inclusion of a random within-breed animal effect was expected because of differences in apparent SNP effects across breeds. The power of this approach to separate across- and within-breed animal effects was limited when allele substitution effects were randomly assigned to QTL, which may explain why adding a within-breed animal effect was not beneficial. For the scenarios for which each QTL explained the same variance, the power to separate both effects might differ, but adding a within-breed animal effect was still not beneficial in terms of accuracy. Using a larger reference population with more animals of each breed may enable to properly separate across-breed animal and within-breed animal effects in a better way, but enlarging reference populations for numerically small breeds is challenging. Thus, to give a conclusive answer about this objective, more data is needed to investigate if it is possible to separate random across-breed and within-breed animal effects, and if this is case, then it is necessary to investigate whether it is beneficial for multi-breed genomic prediction.

## Conclusions

The results of this study show that the accuracy of both single- and multi-breed genomic prediction depends on the properties of the QTL that control the trait. A decrease in average QTL MAF decreased accuracy of genomic prediction, especially when rare alleles had a large effect. Therefore, we demonstrated that the properties of the QTL that control traits (i.e. allele frequency spectra of QTL, distribution of QTL effects) are key parameters that determine the accuracy of both single- and multi-breed genomic predictions. Based on these results, the properties of QTL that underlie a trait can explain the limited benefit or the absence of benefit of combining information from multiple breeds that is described in empirical studies as opposed to the substantial benefit that is achieved in simulation studies. Accuracy of single-, but especially multi-breed genomic prediction, could be increased by using sequence data, since the causative mutations are probably included in the dataset. The results show that the increase in accuracy was consistently, although not significantly, larger when the number of other variants included in the dataset was smaller. Finally, adding a random within-breed animal effect to a GBLUP type of model had no effect on the accuracy of genomic prediction, most likely because the power to separate random across-breed and within-breed animal effects was low.

## Additional files

Additional file 1: Figure S1. Allele frequency distribution of imputed and genotyped variants in Holstein Friesian animals. Description: Figure S1 shows the distribution of allele frequencies of variants with on average a moderately low minor allele frequency (MAF), very low MAF or extremely low MAF in real data and imputed data for Holstein-Friesian animals. Additional file 2: Figure S2. Allele frequency distribution of imputed and genotyped variants in Jersey animals. Description: Figure S2 shows the distribution of allele frequencies of variants with on average a moderately low minor allele frequency (MAF), very low MAF or extremely low MAF in real data and imputed data for Jersey animals. Additional file 3: Figure S3. Allele frequencies of Holstein Friesian versus Jersey animals. Description: Figure S3 shows patterns of allele frequencies for Holstein-Friesian versus Jersey animals. (A) Variants with on average a moderately low minor allele frequency; (B) Variants with on average a very low minor allele frequency; (C) Variants with on average an extremely low minor allele frequency. Additional file 4: Table S1. Average estimated heritabilities for QTL with different properties when 1000 QTL underlie the trait. Description: Table S1 contains average heritabilities (standard errors across replicates) estimated with a model including a random across-breed animal effect and a within-breed animal effect and using 606 384 SNPs to calculate the genomic relationship matrix using different reference populations, different average minor allele frequencies (MAF) of the 1000 QTL that underlie the trait and using simulated allele substitution effects randomly sampled from a gamma distribution (RANDOM) or with each QTL explaining an equal proportion of the genetic variance (VAR). Additional file 5: Figure S4. Accuracies of genomic prediction using different marker densities to calculate the genomic relationship matrix and QTL with very low minor allele frequency. Description: Figure S4 shows average accuracies of genomic prediction (± standard errors) for Holstein-Friesian (HF, solid fill) and Jersey (J, diagonal fill) animals using a model that included a random across-breed animal effect and a within-breed animal effect, seven different reference populations and using simulated allele substitution effects (A) randomly sampled from a gamma distribution or (B) with each QTL explaining an equal proportion of the genetic variance, when 100 QTL underlying the trait were sampled from variants with on average a very low minor allele frequency. The genomic relationship matrices were calculated using 606 384 SNPs (black), 60 000 SNPs (dark grey), 606 384 SNPs plus all sampled QTL (grey), or 60 000 SNPs plus all sampled QTL (light grey). Additional file 6: Figure S5. Accuracies of genomic prediction using different marker densities to calculate the genomic relationship matrix and QTL with extremely low minor allele frequency. Description: Figure S5 shows average accuracies of genomic prediction (± standard errors) for Holstein-Friesian (HF, solid fill) and Jersey (J, diagonal fill) animals using a model that included a random across-breed animal effect and a within-breed animal effect, seven different reference populations and using simulated allele substitution effects (A) randomly sampled from a gamma distribution or (B) with each QTL explaining an equal proportion of the genetic variance, when 100 QTL underlying the trait were sampled from variants with on average an extremely low minor allele frequency. The genomic relationship matrices were calculated using 606 384 SNPs (black), 60 000 SNPs (dark grey), 606 384 SNPs plus all sampled QTL (grey), or 60 000 SNPs plus all sampled QTL (light grey).
