# Supplementary material for: Impact of QTL properties on the accuracy of multi-breed genomic prediction
Source: Genet Sel Evol. 2015 May 8;47(1):42. doi: 10.1186/s12711-015-0124-6 (PMC4424523; doi:10.1186/s12711-015-0124-6)
Supplement: Additional file 1: Figure S1. — Allele frequency distribution of imputed and genotyped variants in Holstein Friesian animals. Description: Figure S1 shows the distribution of allele frequencies of variants with on average a moderately low minor allele frequency (MAF), very low MAF or extremely low MAF in real data and imputed data for Holstein-Friesian animals. [file 12711_2015_124_MOESM1_ESM.pdf]

Additional file 1: Figure S1

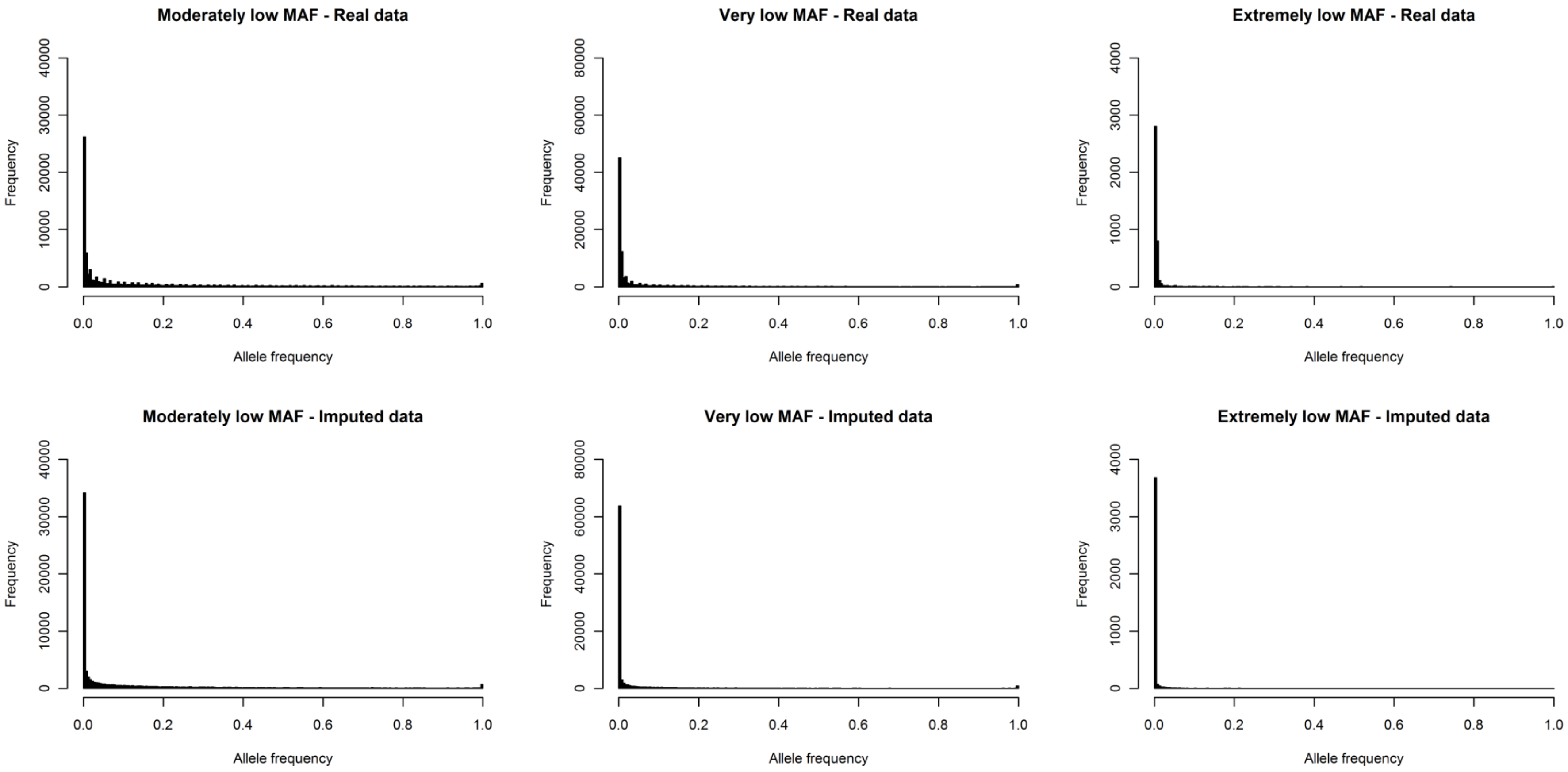

Figure S1 – Allele frequency distribution of imputed and genotyped variants in Holstein Friesian animals. Distribution of allele frequencies of variants with on average a moderately low minor allele frequency (MAF), very low MAF or extremely low MAF in real data and imputed data for Holstein Friesian animals.
