# Supplementary material for: Impact of QTL properties on the accuracy of multi-breed genomic prediction
Source: Genet Sel Evol. 2015 May 8;47(1):42. doi: 10.1186/s12711-015-0124-6 (PMC4424523; doi:10.1186/s12711-015-0124-6)
Supplement: Additional file 2: Figure S2. — Allele frequency distribution of imputed and genotyped variants in Jersey animals. Description: Figure S2 shows the distribution of allele frequencies of variants with on average a moderately low minor allele frequency (MAF), very low MAF or extremely low MAF in real data and imputed data for Jersey animals. [file 12711_2015_124_MOESM2_ESM.pdf]

Additional file 2

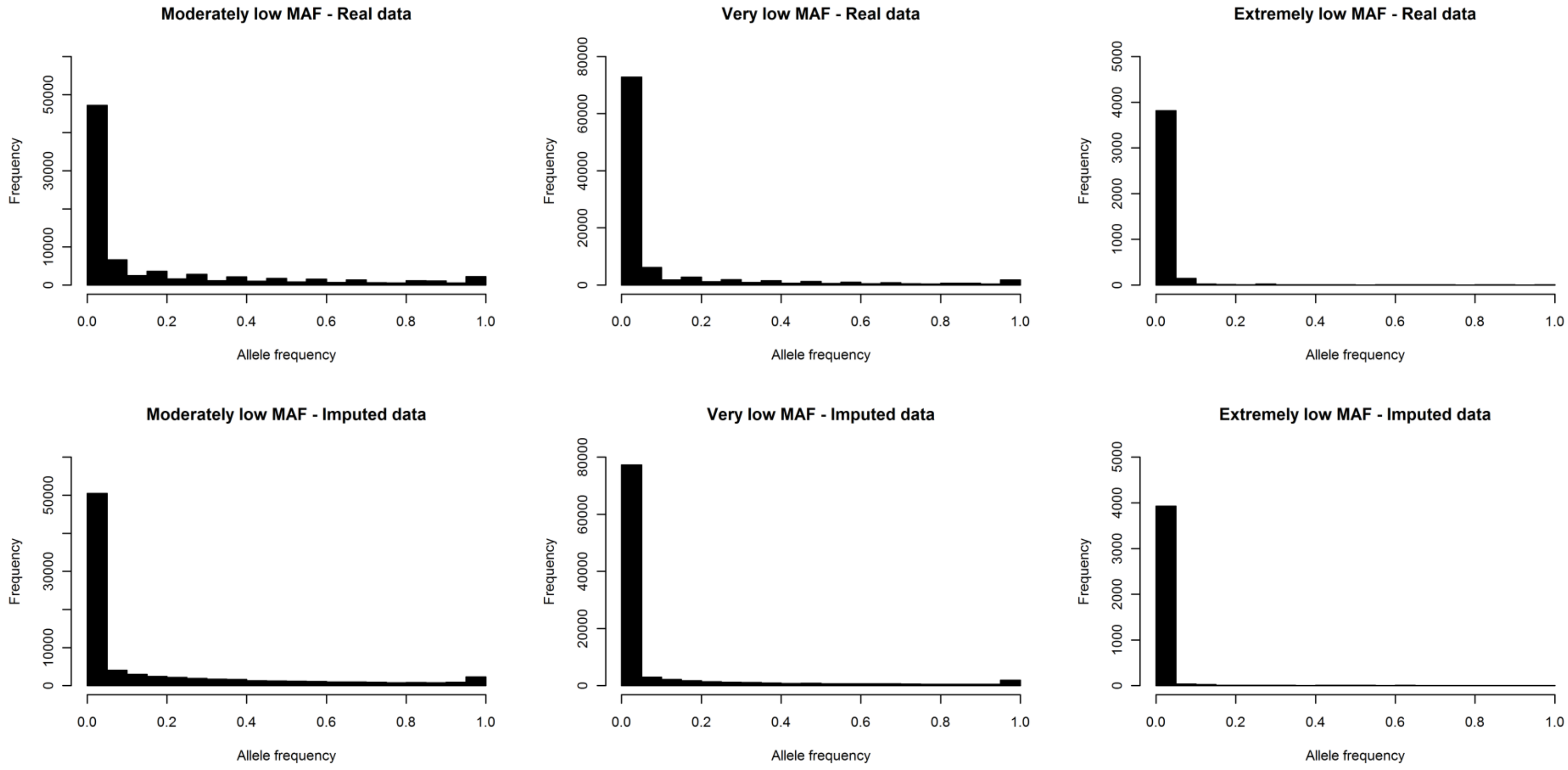

Figure S2 - Allele frequency distribution of imputed and genotyped variants in Jersey animals. Distribution of allele frequencies of variants with on average a moderately low minor allele frequency (MAF), very low MAF or extremely low MAF in real data and imputed data for Jersey animals.
