# Supplementary material for: Impact of QTL properties on the accuracy of multi-breed genomic prediction
Source: Genet Sel Evol. 2015 May 8;47(1):42. doi: 10.1186/s12711-015-0124-6 (PMC4424523; doi:10.1186/s12711-015-0124-6)
Supplement: Additional file 3: Figure S3. — Allele frequencies of Holstein Friesian versus Jersey animals. Description: Figure S3 shows patterns of allele frequencies for Holstein-Friesian versus Jersey animals. (A) Variants with on average a moderately low minor allele frequency; (B) Variants with on average a very low minor allele frequency; (C) Variants with on average an extremely low minor allele frequency. [file 12711_2015_124_MOESM3_ESM.pdf]

Additional file 3

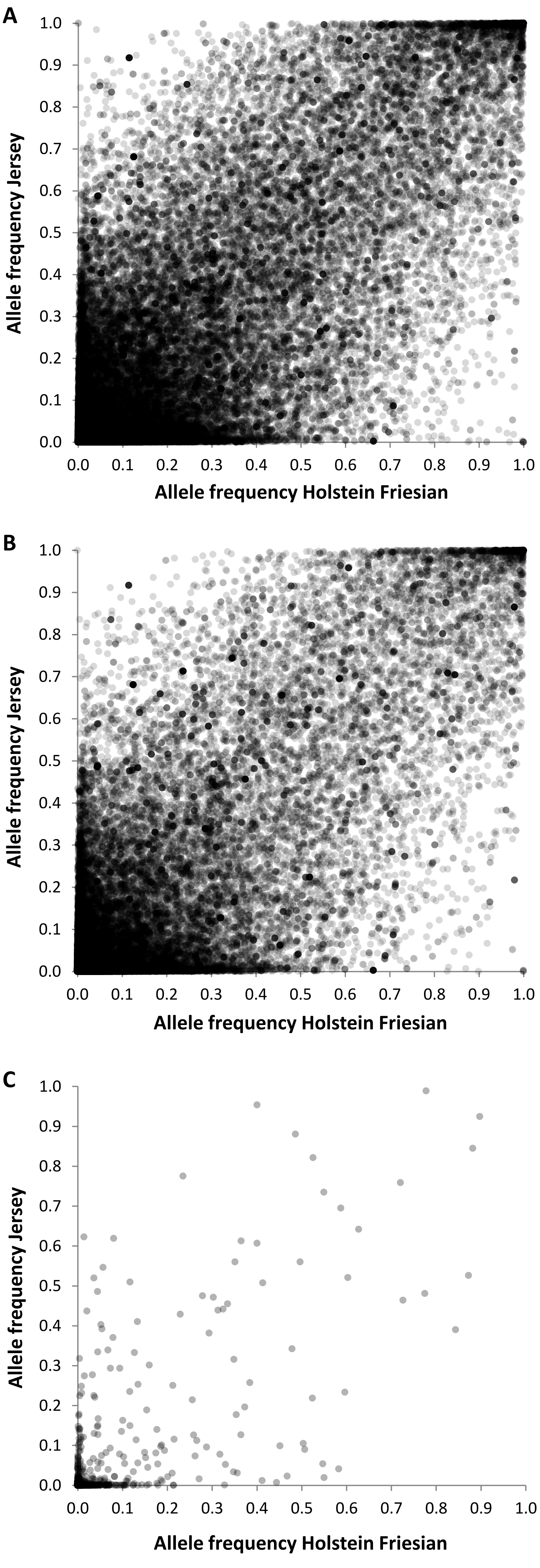

Figure S3 - Allele frequencies of Holstein Friesian versus Jersey animals. Patterns of allele frequencies for Holstein Friesian versus Jersey animals. (A) Variants with on average a moderately low minor allele frequency; (B) Variants with on average a very low minor allele frequency; (C) Variants with on average an extremely low minor allele frequency.
