# Supplementary material for: Impact of QTL properties on the accuracy of multi-breed genomic prediction
Source: Genet Sel Evol. 2015 May 8;47(1):42. doi: 10.1186/s12711-015-0124-6 (PMC4424523; doi:10.1186/s12711-015-0124-6)
Supplement: Additional file 4: Table S1. — Average estimated heritabilities for QTL with different properties when 1000 QTL underlie the trait. Description: Table S1 contains average heritabilities (standard errors across replicates) estimated with a model including a random across-breed animal effect and a within-breed animal effect and using 606 384 SNPs to calculate the genomic relationship matrix using different reference populations, different average minor allele frequencies (MAF) of the 1000 QTL that underlie the trait and using simulated allele substitution effects randomly sampled from a gamma distribution (RANDOM) or with each QTL explaining an equal proportion of the genetic variance (VAR). [file 12711_2015_124_MOESM4_ESM.pdf]

Additional file 4

Table S1 – Average estimated heritabilities for QTL with different properties when 1000 QTL underlie the trait. Average heritabilities (standard errors across replicates) estimated with a model including a random across-breed animal effect and a within-breed animal effect and using 606 384 SNPs to calculate the genomic relationship matrix using different reference populations, different average minor allele frequencies (MAF) of the 1000 QTL that underlie the trait and using simulated allele substitution effects randomly sampled from a gamma distribution (RANDOM) or with each QTL explaining an equal proportion of the genetic variance (VAR).

			RANDOM						VAR					
			Moderately low MAF[3]		Very low MAF[3]		Extremely low MAF[3]		Moderately low MAF[3]		Very low MAF[3]		Extremely low MAF[3]	
**Scenarios**	**Nb HF[1]**	**Nb J[2]**												
**1**	**2 000**	**2 000**	0.79	(0.020)	0.77	(0.021)	0.72	(0.023)	0.59	(0.028)	0.45	(0.031)	0.21	(0.031)
**2**	**2 000**	**500**	0.80	(0.030)	0.76	(0.032)	0.72	(0.033)	0.54	(0.040)	0.39	(0.042)	0.16	(0.037)
**3**	**2 000**	**100**	0.80	(0.032)	0.76	(0.035)	0.71	(0.036)	0.54	(0.043)	0.39	(0.045)	0.15	(0.039)
**4**	**2 000**	**0**	0.80	(0.033)	0.75	(0.035)	0.70	(0.037)	0.55	(0.044)	0.39	(0.046)	0.15	(0.036)
**5**	**500**	**2 000**	0.78	(0.025)	0.77	(0.025)	0.73	(0.028)	0.61	(0.034)	0.49	(0.039)	0.26	(0.041)
**6**	**100**	**2 000**	0.78	(0.026)	0.78	(0.026)	0.72	(0.030)	0.58	(0.037)	0.46	(0.041)	0.23	(0.042)
**7**	**0**	**2 000**	0.78	(0.027)	0.78	(0.027)	0.73	(0.030)	0.56	(0.038)	0.45	(0.042)	0.23	(0.043)

[1] Nb HF = Number of Holstein Friesian animals

[2] Nb J = Number of Jersey animals

[3] MAF = minor allele frequency
