# Supplementary material for: Impact of QTL properties on the accuracy of multi-breed genomic prediction
Source: Genet Sel Evol. 2015 May 8;47(1):42. doi: 10.1186/s12711-015-0124-6 (PMC4424523; doi:10.1186/s12711-015-0124-6)
Supplement: Additional file 5: Figure S4. — Accuracies of genomic prediction using different marker densities to calculate the genomic relationship matrix and QTL with very low minor allele frequency. Description: Figure S4 shows average accuracies of genomic prediction (± standard errors) for Holstein-Friesian (HF, solid fill) and Jersey (J, diagonal fill) animals using a model that included a random across-breed animal effect and a within-breed animal effect, seven different reference populations and using simulated allele substitution effects (A) randomly sampled from a gamma distribution or (B) with each QTL explaining an equal proportion of the genetic variance, when 100 QTL underlying the trait were sampled from variants with on average a very low minor allele frequency. The genomic relationship matrices were calculated using 606 384 SNPs (black), 60 000 SNPs (dark grey), 606 384 SNPs plus all sampled QTL (grey), or 60 000 SNPs plus all sampled QTL (light grey). [file 12711_2015_124_MOESM5_ESM.pdf]

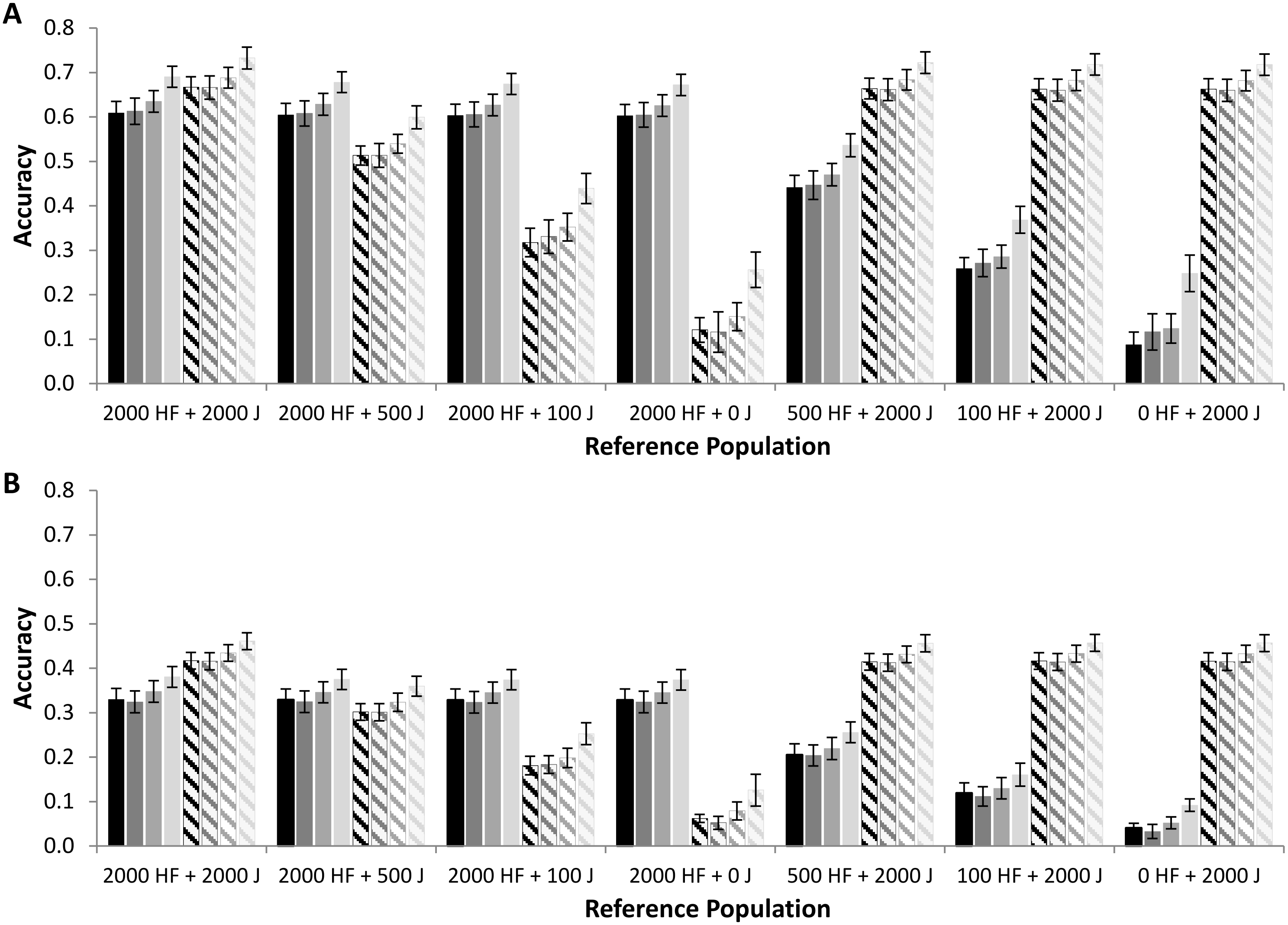

Figure S4 - Accuracies of genomic prediction using different marker densities to calculate the genomic relationship matrix and QTL with very low minor allele frequency. Average accuracies of genomic prediction (± standard errors) for Holstein-Friesian (HF, solid fill) and Jersey (J, diagonal fill) animals using a model that included a random across-breed animal effect and a within-breed animal effect, seven different reference populations and using simulated allele substitution effects (A) randomly sampled from a gamma distribution or (B) with each QTL explaining an equal proportion of the genetic variance, when 100 QTL underlying the trait were sampled from variants with on average a very low minor allele frequency. The genomic relationship matrices were calculated using 606 384 SNPs (black), 60 000 SNPs (dark grey), 606 384 SNPs plus all sampled QTL (grey), or 60 000 SNPs plus all sampled QTL (light grey).
